# Supplementary material for: Sex Differences in Coronary Artery Disease and Diabetes Revealed by scRNA-Seq and CITE-Seq of Human CD4+ T Cells
Source: Int J Mol Sci. 2022 Aug 30;23(17):9875. doi: 10.3390/ijms23179875 (PMC9456056; doi:10.3390/ijms23179875)
Supplement: Supplementary file 1 [file ijms-23-09875-s001.zip › ijms-1884353-SI.zip!/ijms-1884353-SI.pdf]

Supplemental Materials

Ryosuke Saigusa[1], Jenifer Vallejo[1], Rishab Gulati[1], Sujit Silas Armstrong Suthahar[1], Vasantika Suryawanshi[1], Ahmad Alimadadi[1], Jeffrey Makings[1], Christopher P. Durant[1], Antoine Freuchet[1], Payel Roy[1], Yanal Ghosheh[1], William Pandori[1], Tanyaporn Pattarabanjird[2],Fabrizio Drago[2], Angela Taylor[2], Coleen A. McNamara[2], Avishai Shemesh[3], Lewis L. Lanier[3], Catherine C. Hedrick[1], Klaus Ley[1,4]

[1]La Jolla Institute for Immunology, La Jolla, CA, USA.
[2]Cardiovascular Research Center, Cardiovascular Division, Department of Medicine, University of Virginia, Charlottesville.
[3]Parker Institute for Cancer Immunotherapy, University of California, San Francisco, CA, USA; Department of Microbiology and Immunology, University of California, San Francisco, CA, USA.
[4]University of California San Diego, San Diego, CA, USA.

*Corresponding Author
Klaus Ley, MD
La Jolla Institute for Immunology
9420 Athena Circle
La Jolla, CA 92037, USA
(858) 752-6661 (tel)
(858) 752-6985 (fax)
klaus@lji.org

Content

Supplemental Table 1-6 (Table S1-6)
Supplemental Figure 1-5 (Figure S1-5)
Legends of Supplemental Excel File 1-4

Table S1. All reagents, manufacturers and catalogue numbers

Reagent	Vendor	Catalogue #
cRPMI[*1]		-
PBS	Fisher Scientific	10010049
FBS	Gemini	100106-500mL
Trypan Blue	Thermo Fisher	15250061
Fc Block	BD	564220
SMK	BD	633781
DRAQ7	BD	564904
Calcein AM[*2]	Thermo Fisher	C1430
DMSO	Thermo Fisher	D12345
AbSeq	BD	-
Rhapsody Reagent Kit		633771
Rhapsody Human Immune Response Panel		633750
Rhapsody Custom Reagent Panel		633742
AMPure XP beads	Beckman Coulter	A63881
Ethanol	Milipore Sigma	E7023-500ml
Nuclease Free Water	Qiagen	129114
D1000 ScreenTape	Agilent	5067-5584
D1000 Sample Buffer	Agilent	5067-5603
Qubit Reagent Kit	Thermo Fisher	Q33231
NovaSeq S1 100 Cycle Kit	Illumina	20012865
NovaSeq S2 100 Cycle Kit	Illumina	20012862

*1: From 500mL of RPMI-1640 (with L-Glutamine) remove 75.5mL and transfer to two 50mL conical tubes for future potential use. Add 50 mL of 100% Human Serum Albumin (HSA). Add 5mL of the following (concentrations indicated are of stock solutions): HEPES (1M), Sodium pyruvate (100X) MEM-NEAA (100X), Pen Strep (Penicillin-Streptomycin), GlutaMAX Add 0.5mL of Mercaptoethanol (1000X). Mix by inversion in RPMI-1640 original bottle. Carefully transfer solution to a 500mL CorningStore at 4°C. Vacuum filter system with a 0.45mm filter size. *2: Resuspended in DMSO.

Table S2. The viability of each sample tube

Sample #	Average	Sample #	Average
1	92.50%	34	77.30%
2	90.00%	36	91.38%
3	84.00%	37	90.79%
4	89.00%	38	87.86%
6	93.00%	39	84.09%
7	90.50%	40	90.06%
8	93.00%	41	90.09%
9	90.00%	42	95.53%
10	94.79%	43	93.23%
11	91.75%	44	87.69%
12	93.10%	45	97.40%
13	94.47%	46	95.17%
14	89.55%	47	84.85%
15	84.97%	48	88.61%
16	92.13%	49	92.62%
17	93.62%	50	92.39%
18	90.33%	51	89.20%
19	90.72%	52	94.69%
20	93.14%	53	89.34%
21	91.92%	54	93.21%
22	93.43%	55	90.28%
23	90.45%	56	93.56%
24	94.54%	57	98.80%
25	90.02%	59	95.65%
26	88.50%	60	94.42%
27	82.70%	61	93.10%
28	92.47%	62	93.61%
29	89.52%	64	95.96%
30	88.70%	65	83.35%
31	89.74%	**Min**	77.1%
32	77.10%	**Max**	98.8%
33	89.26%	**Median**	90.8%
		**Average**	90.7%

Table S3. The information of 51 AbSeq antibodies

Specificity	Clone	Catalogue Number
CD11b	M1/70	940008
CD11c	B-LY6	940024
CD123 (IL-3RA)	7G3	940020
CD126 (IL-6R)	M5	940090
CD127 (IL-7R)	HIL-7R-M21	940012
CD137	4B4-1	940055
CD14	MPHIP9	940005
CD141	1A4	940079
CD142	HTF-1	940280
CD152 (CTLA-4)	BNI3	940034
CD154	TRAP1	940053
CD16	3G8	940006
CD163	GHI/61	940058
CD183 (CXCR3)	1C6/CXCR3	940030
CD184 (CXCR4)	12G5	940056
CD185 (CXCR5)	RF8B2	940042
CD19	SJ25C1	940004
CD192 (CCR2)	1D9	940286
CD194 (CCR4)	1G1	940047
CD195 (CCR5)	2D7/CCR5	940050
CD196 (CCR6)	11A9	940033
CD197 (CCR7)	3D12	940014
CD2	RPA-2.10	940046
CD20	2H7	940016
CD206	19.2	940068
CD223 (LAG-3)	T47-530	940080
CD25	2A3	940009
CD27	M-T271	940018
CD3	SK7	940000
CD36	CB38 (NL07)	940224
CD38	HIT2	940013
CD4	SK3	940001
CD45RA	HI100	940011
CD45RO	UCHL1	940022

CD56	NCAM16.2	940007
CD69	FN50	940019
CD8	RPA-T8	940003
CD86	2331(FUN-1)	940025
CD9	M-L13	940078
HLA-DR (CD74)	G46-6	940010
CD24	ML5	940028
CD33	WM53	940031
IgM	G20-127	940276
IgD	IA6-2	940026
CD43	1G10	940728
CD273	MIH18	746072
CD274	MIH1	940035
CD95	DX2	940037
CD279	MIH4	940467
TLR4-APC	610015	not applicable
SLAN-PE	M-DC8	not applicable
PE	E31-1459	460077
APC	E30-221	460078

Table S4. Thresholds of each antibody expression.

Antibody	threshold
**CD2**	2.4
**CD3**	2
**CD4**	1.65
**CD8**	3.7
**CD9**	1.3
**CD11b**	removed from all the analysis
**CD11c**	2
**CD14**	1.65
**CD16**	3.25
**CD19**	1.3
**CD20**	0.45
**CD24**	0.6
**CD25**	not used for clustering
**CD27**	1.15
**CD33**	1.3
**CD36**	3.4
**CD38**	1
**CD43**	2
**CD45RA**	2.15
**CD45RO**	0.8
**CD56**	0.5
**CD69**	not used for clustering
**CD86**	0.9
**CD95**	1.85
**CD123**	1

Antibody	threshold
**CD126**	not used for clustering
**CD127**	0.8
**CD137**	not used for clustering
**CD141**	0.5
**CD142**	not used for clustering
**CD152**	not used for clustering
**CD154**	removed from all the analysis
**CD163**	not used for clustering
**CD183**	1.1
**CD184**	not used for clustering
**CD185**	0.2
**CD192**	1.25
**CD194**	not used for clustering
**CD195**	not used for clustering
**CD196**	not used for clustering
**CD197**	not used for clustering
**CD206**	not used for clustering
**CD223**	not used for clustering
**CD273 PDL2**	not used for clustering
**CD274 PDL1**	1
**CD279 PD1**	0.75
**CD74**	1.6
**IgD**	1.5
**IgM**	0.8
**SA06 TLR4**	0.1
**SA16 SLAN**	0.37

Table S5A-E. Clinical statistics of all the samples.

A. CAD Low vs CAD High in Diabetic/Non-Diabetic Men on Statins and Not Diabetic Men not on Statins.

	**Diabetic Men on Statins (n=9/8 per group)**			**Non-Diabetic Men on Statins (n=9/8 per group)**			**Not Diabetic Men not on Statins (n=5 per group)**		
**Variable** Count [%] or Median [± SD]	**CAD Low Gensini < 6** (n=9)	**CAD High Gensini > 30** (n=8)	**P-value †**	**CAD Low Gensini < 6** (n=8)	**CAD High Gensini > 30** (n=9)	**P-value†**	**CAD Low Gensini < 6** (n=5)	**CAD High Gensini > 30** (n=5)	**P-value†**
**General Characteristics**									
Age (years)	62 [±8.48]	63 [±10.29]	0.7	65 [±6.23]	64 [±6.73]	0.92	63 [±12.97]	64 [±13.40]	0.92
Race (Caucasian)	9 [100%]	8 [100%]		8 [100%]	8 [88.9%]	0.33	3 [60%]	5 [100%]	0.12
Ethnicity (Non-Hispanic)	9 [100%]	8 [100%]		8 [100%]	7 [77.78%]	0.15	4 [80%]	5 [100%]	0.29
Current Smoker (Yes)	0 [0%]	0 [0%]		0 [0%]	0 [0%]		0 [0%]	0 [0%]	
Former Smoker (Yes)	5 [55.6%]	5 [62.5%]	0.77	4 [50%]	3 [33.3%]	0.49	3 [60%]	3 [60%]	
BMI	35 [±6.51]	32 [±6.86]	0.54	32 [±7.61]	31 [±5.76]	0.51	30 [±5.13]	29 [±5.72]	0.68
BP Systolic	147 [±19.76]	132 [±18.99]	0.12	138 [±18.96]	141 [±5.94]	0.33	123 [±13.83]	143 [±12.63]	0.07
BP Diastolic	80 [±10.49]	70 [±12.67]	0.12	81 [±15.54]	78 [±13.13]	0.6	79 [±13.12]	83 [±17.08]	0.92
**Medications**									
Diuretics (Yes)	0 [0%]	2 [25%]	0.11	1 [12.5%]	4 [44.4%]	0.15	0 [0%]	1 [20%]	0.29
Beta Blockers (Yes)	7 [77.8%]	5 [62.5%]	0.49	4 [50%]	4 [44.4%]	0.82	2 [40%]	2 [40%]	

Calcium Channel Blockers (Yes)	1 [11.1%]	1 [12.5%]	0.93	2 [25%]	2 [22.2%]	0.89	0 [0%]	0 [0%]	
ACE (Yes)	4 [44.4%]	4 [50%]	0.82	2 [25%]	2 [22.2%]	0.89	1 [20%]	1 [20%]	
ATR (Yes)	2 [22.2%]	1 [12.5%]	0.6	1 [12.5%]	0 [0%]	0.27	1 [20%]	0 [100%]	0.29
NSAID (Yes)	8 [88.9%]	7 [87.5%]	0.93	7 [87.5%]	9 [100%]	0.27	3 [60%]	4 [80%]	0.49
**Lab Values**									
Creatinine	0.66 [±0.35]	0.85 [±0.17]	0.34	0.95 [±0.18]	0.99 [±0.24]	0.85	0.84 [±0.15]	0.82 [±0.11]	0.99
Hs-CRP	1.54 [±1.30]	2.18 [±1.98]	0.48	2.25 [±2.45]	2.98 [±3.34]	0.82	4.06 [±5.60]	1.77 [±2.14]	0.84
Total Cholesterol (mg/dL)	134 [±30.62]	135 [±35.38]	0.96	129 [±22.12]	140 [±40.03]	0.6	150 [±27.33]	169 [±57.09]	0.84
Triglyceride (mg/dL)	146 [±100.08]	162 [±94.74]	0.54	125 [±53.86]	103 [±64.02]	0.43	84 [±39.23]	89 [±12.46]	0.43
HDL Cholesterol (mg/dL)	38 [±9.42]	36 [±10.31]	0.6	39 [±15.82]	43 [±10.39]	0.33	42 [±8.53]	40 [±8.35]	0.76
LDL Cholesterol (mg/dL)	72 [±28.29]	72 [±31.61]	0.89	69 [±10.97]	80 [±29.89]	0.6	94 [±24.97]	114 [±48.43]	0.69
Glucose (mg/dL)	151 [±60.02]	144 [±36.64]	0.74	98 [±11.93]	94 [±13.36]	0.81	95 [±13.87]	101 [±6.91]	0.76
A1c (%)	7.31 [±1.46]	7.18 [±1.31]	0.81	5.81 [±0.76]	5.54 [±0.44]	0.85	5.50 [±0.48]	5.67 [±0.17]	0.92
**Disease Severity**									
Gensini Scores	1.83 [±1.97]	66.19 [±31.75]	0	3.00 [±2.67]	70.33 [37.52]	0	1.10 [±2.46]	81.80 [±32.45]	0.03

†Categorical variables were calculated by Chi-square test and continuous variables by Mann Whitney

B. CAD Low vs CAD High in Women on Statins with and without diabetes.

Variable<br>Count [%] or Median [± SD]	CAD Low<br>Gensini < 6<br>(n=7)	CAD High<br>Gensini > 30<br>(n=9)	P-value†
**General Characteristics**			
Age (years)	65 [±7.72]	67 [±8.82]	0.47
Race (Caucasian)	7 [100%]	9 [100%]	0.24
Ethnicity (Non-Hispanic)	6 [85.7%]	9 [100%]	0.24
Diabetes (Yes)	4 [57.1%]	5 [55.6%]	0.95
Current Smoker (Yes)	2 [28.6%]	3 [33.3%]	0.84
Former Smoker (Yes)	4 [57.1%]	2 [22.2%]	0.15
BMI	39 [±4.94]	29 [±6.26]	0.03
BP Systolic	124 [±16.78]	150 [±26.29]	0.08
BP Diastolic	71 [±10.92]	77 [±14.67]	0.44
**Medications**			
Diuretics (Yes)	3 [42.86%]	4 [44.4%]	0.94
Beta Blockers (Yes)	5 [71.43%]	5 [55.6%]	0.52
Calcium Channel Blockers (Yes)	1 [14.3%]	5 [55.6%]	0.09
ACE (Yes)	4 [57.14%]	4 [44.4%]	0.61
ATR (Yes)	0 [100%]	0 [100%]	
NSAID (Yes)	5 [71.4%]	7 [77.8%]	0.77
**Lab Values**			
Creatinine	0.81 [±0.13]	0.70 [±0.17]	0.18
Hs-CRP	6.25 [±4.14]	3.17 [±2.42]	0.07
Total Cholesterol (mg/dL)	159 [±19.70]	163 [±55.38]	0.53
Triglyceride (mg/dL)	178 [±69.92]	101 [±49.50]	0.05
HDL Cholesterol (mg/dL)	41 [±10.09]	54 [±20.35]	0.17
LDL Cholesterol (mg/dL)	88 [±21.58]	92 [±41.28]	0.99
Glucose (mg/dL)	128 [±40.23]	111 [±23.31]	0.53
A1c (%)	7.3 [±2.12]	6.36 [±0.62]	0.68
**Disease Severity**			
Gensini Scores	3.00 [±2.81]	39.22 [±7.55]	0.005

C. CAD Low vs CAD High in all the participants

Variable<br>Count [%] or Mean [± SD]	CAD Low (n=29)	CAD High (n=32)	p-value
**Demographics**			
Age (years)	64 [±8.27]	65 [±9.12]	0.41
Sex (Male)	22 (76%)	22 (69%)	0.54
Race (Caucasian)	27 (93%)	31 (97%)	0.50
Ethnicity (Non-Hispanic)	27 (93%)	30 (94%)	0.92
Diabetes (Yes)	13 (45%)	14 (44%)	0.93
Smoking	18 (62%)	16 (50%)	0.34
BMI	34.2 [±6.65]	30.6 [±6.02]	0.04
BP Systolic	135 [±19.85]	143 [±19.36]	0.09
BP Diastolic	78 [±12.56]	76 [±13.97]	0.57
**Medications**			
Statins (Yes)	24 (83%)	26 (81%)	0.88
Diuretics (Yes)	4 (14%)	11 (34%)	0.06
Beta Blockers (Yes)	18 (62%)	16 (50%)	0.34
Calcium Channel Blockers (Yes)	4 (14%)	9 (28%)	0.17
ACE (Yes)	11 (38%)	11 (34%)	0.77
ATR (Yes)	4 (14%)	2 (6%)	0.32
NSAID (Yes)	23 (79%)	28 (88%)	0.39
**Lab Values**			
Creatinine	0.81 [±0.25]	0.84 [±0.20]	0.84
Hs-CRP	3.3 [±3.71]	2.64 [±2.48]	0.78
Total Cholesterol (mg/dL)	141 [±27.01]	152 [±46.99]	0.65
Triglyceride (mg/dL)	137 [±76.39]	114 [±67.66]	0.19
HDL Cholesterol (mg/dL)	40 [±11.05]	44 [±14.73]	0.36
LDL Cholesterol (mg/dL)	79 [±23.49]	89 [±38.98]	0.60
Glucose (mg/dL)	121 [±44.74]	113 [±29.46]	0.98
A1c (%)	6.6 [±1.56]	6.2 [±0.98]	0.81
**Disease Severity**			
Gensini Scores	2.3 [±2.46]	61.3 [±31.66]	<.0001

D. Diabetes vs No Diabetes in all the participants

Variable<br>Count [%] or Mean [± SD]	Diabetes (n=27)	No Diabetes (n=34)	p-value
**Demographics**			
Age (years)	65 [±8.46]	64 [±8.98]	0.87
Sex (Male)	17 (63%)	27 (79%)	0.15
Race (Caucasian)	27 (100%)	31 (91%)	0.11
Ethnicity (Non-Hispanic)	27 (100%)	30 (88%)	0.07
Smoking (Yes)	17 (63%)	17 (50%)	0.59
BMI	33.5 [±6.46]	31.4 [±6.53]	0.25
BP Systolic	142 [±24.62]	136 [±14.91]	0.52
BP Diastolic	75 [±11.66]	79 [±14.23]	0.31
**Medications**			
Statins (Yes)	26 (96%)	24 (71%)	0.01
Diuretics (Yes)	6 (22%)	9 (26%)	0.70
Beta Blockers (Yes)	18 (67%)	16 (47%)	0.13
Calcium Channel Blockers (Yes)	6 (22%)	7 (21%)	0.88
ACE (Yes)	13 (48%)	9 (26%)	0.08
ATR (Yes)	4 (15%)	2 (6%)	0.24
NSAID (Yes)	24 (89%)	27 (79%)	0.32
**Lab Values**			
Creatinine	0.73 [±0.24]	0.90 [±0.18]	0.007
Hs-CRP	8.3 [±28.42]	3.1 [±3.59]	0.48
Total Cholesterol (mg/dL)	143 [±36.43]	150 [±40.95]	0.52
Triglyceride (mg/dL)	153 [±87.38]	103 [±48.21]	0.03
HDL Cholesterol (mg/dL)	40 [±14.77]	43 [±11.80]	0.16
LDL Cholesterol (mg/dL)	78 [±30.83]	90 [±33.60]	0.26
Glucose (mg/dL)	141 [±43.71]	98 [±14.15]	<.0001
A1c (%)	7.3 [±1.41]	5.7 [±0.50]	<.0001
**Disease Severity**			
Gensini Scores	29.4 [±32.84]	36.28 [±40.95]	0.69

E. Men vs Women in all the participants

Variable<br>Count [%] or Mean [± SD]	Men (n=44)	Women (n=17)	p-value
**Demographics**			
Age (years)	64 [±8.86]	67 [±8.04]	0.24
Race (Caucasian)	41 (93%)	17 (100%)	0.27
Ethnicity (Non-Hispanic)	41 (93%)	16 (94%)	0.89
Diabetes (Yes)	17 (39%)	10 (59%)	0.15
Smoking (Yes)	23 (52%)	11 (65%)	0.38
BMI	32.9 [±6.33]	33.4 [±7.10]	0.37
BP Systolic	138 [±16.86]	141 [±26.53]	0.91
BP Diastolic	78 [±13.44]	75 [±12.72]	0.30
**Medications**			
Statins (Yes)	34 (77%)	16 (94%)	0.13
Diuretics (Yes)	8 (18%)	7 (41%)	0.06
Beta Blockers (Yes)	24 (55%)	10 (59%)	0.76
Calcium Channel Blockers (Yes)	6 (14%)	7 (41%)	0.02
ACE (Yes)	14 (32%)	8 (47%)	0.27
ATR (Yes)	5 (11%)	1 (6%)	0.52
NSAID (Yes)	38 (86%)	13 (76%)	0.35
**Lab Values**			
Creatinine	0.85 [±0.24]	0.75 [±0.15]	0.04
Hs-CRP	2.4 [±2.82]	4.4 [±3.44]	0.001
Total Cholesterol (mg/dL)	140 [±35.70]	164 [±42.58]	0.05
Triglyceride (mg/dL)	123 [±74.17]	131 [±68.80]	0.54
HDL Cholesterol (mg/dL)	39 [±10.68]	48 [±16.93]	0.06
LDL Cholesterol (mg/dL)	81 [±31.30]	94 [±35.20]	0.16
Glucose (mg/dL)	116 [±39.98]	118 [±30.93]	0.41
A1c (%)	6.3 [±1.21]	6.8 [±1.45]	0.06
**Disease Severity**			
Gensini Scores	36.8 [±42.13]	24.1 [±19.05]	0.58

Table S6. The 31 overlapped genes significantly upregulated in both CAD and DM

LAIR2	Leukocyte-Associated Immunoglobulin-Like Receptor 2, CD306
LTB	lymphotoxin β
TCF7	TCF1, exhaustion marker. necessary for T cell differentiation
GNAI2	G Protein Subunit Alpha I2
R3HDM4	nucleic acid binding protein
CTSW	Cathepsin W, regulation of T-cell cytolytic activity
CD7	immunoglobulin family, T-B cell interactions
STAT6	Signal Transducer And Activator Of Transcription 6, IL-4 signaling
IFITM3	IFN-induced antiviral protein, disrupts intracellular cholesterol homeostasis
TNFRSF25	Death Receptor 3 (DR3) for TWEAK. Activates NF-kappa-B, apoptosis
PXN	paxillin, actin-membrane attachment at sites of cell adhesion
XPO6	exportin6, mediates nuclear export of actin and profilin-actin complexes
SRGN	serglycin, cell granule proteoglycan
LGALS9	Galectin 9, S-type lectin for β-Gal, binds HAVCR2 to induce Th1 death
S100A12	binds calcium, zinc, copper. Binds to AGE receptor, pro-inflammatory
CD3E	εsubunit of CD3
ZAP70	TCR signaling, homologue of SYK
CD4	co-receptor for MHC-II
TSPAN32	tetraspanin associated with malignancies
ITGB2	β2 integrin, CD18
IGBP1	immunoglobulin binding protein-1. regulates phosphatases PP2A, PP4, 6
JUNB	JUN-B proto-oncogene, subunit of AP-1
CD37	tetraspanin, complexes with integrins, T-B cell interactions
CTSD	cathepsin D protease, activates hormones and growth factors
CD6	adhesion molecule, binds ALCAM/CD166
CD3G	γ subunit of CD3
BIN2	Modulates membrane curvature and mediates membrane tubulation
CD52	CAMPATH-1 antigen, carries and orients glycans
CD74	Stabilizes empty MHC class II alpha/beta heterodimers
CD27	TNFRSF7. Required for generation and maintenance of T cell immunity
NKG7	Natural Killer Cell Granule Protein 7, in granule membrane

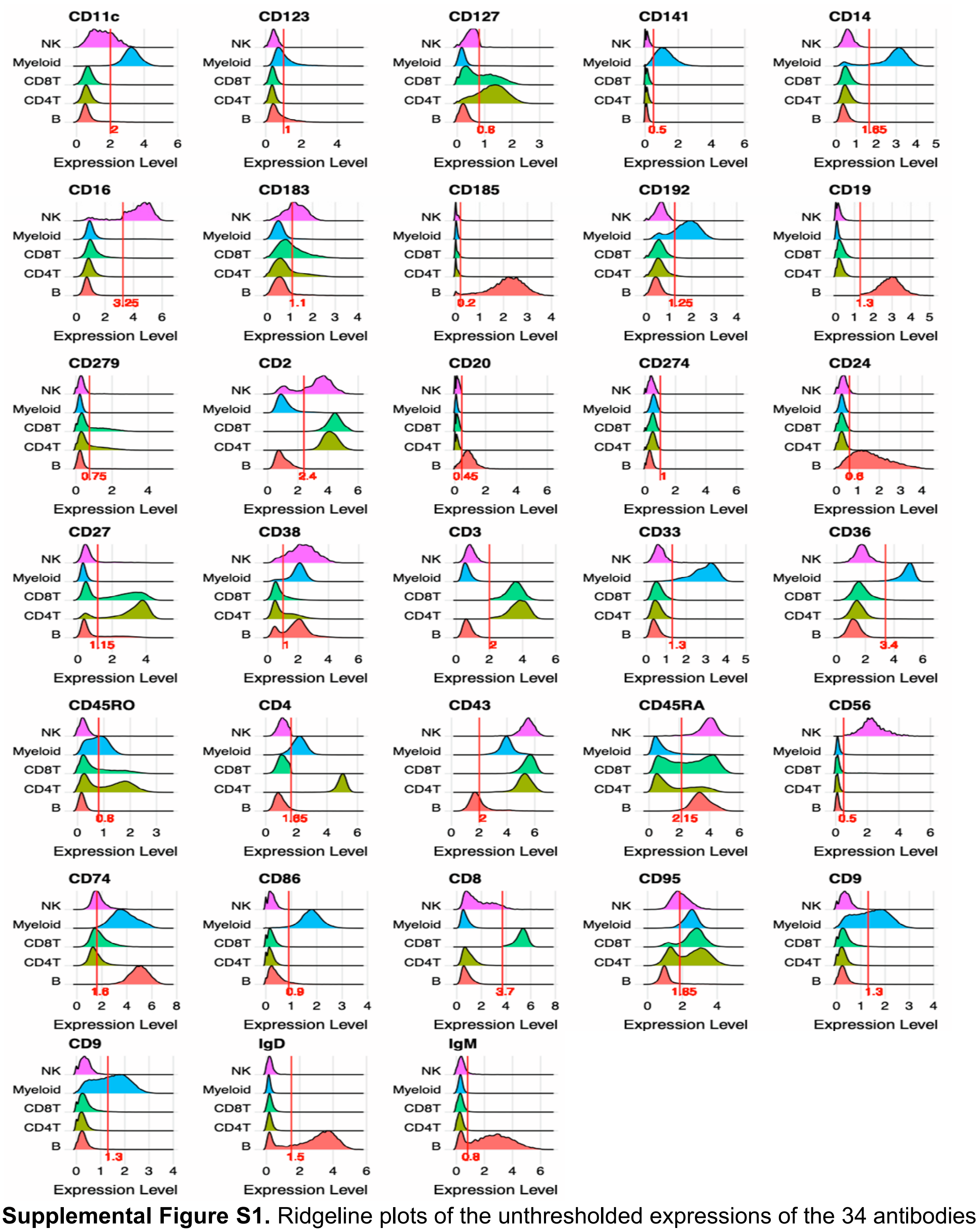

Supplemental Figure S1. Ridgeline plots of the unthresholded expressions of the 34 antibodies used for clustering for each main cell types (CD4 T cells, CD8 T cells, myeloid cells, NK cells and B cells) using Seurat. These plots separately show the distribution of each value of CLR normalized antibody derived tag for each main cell type. Expression levels are shown on x-axis. Thresholding lines are shown in black line in each plot.

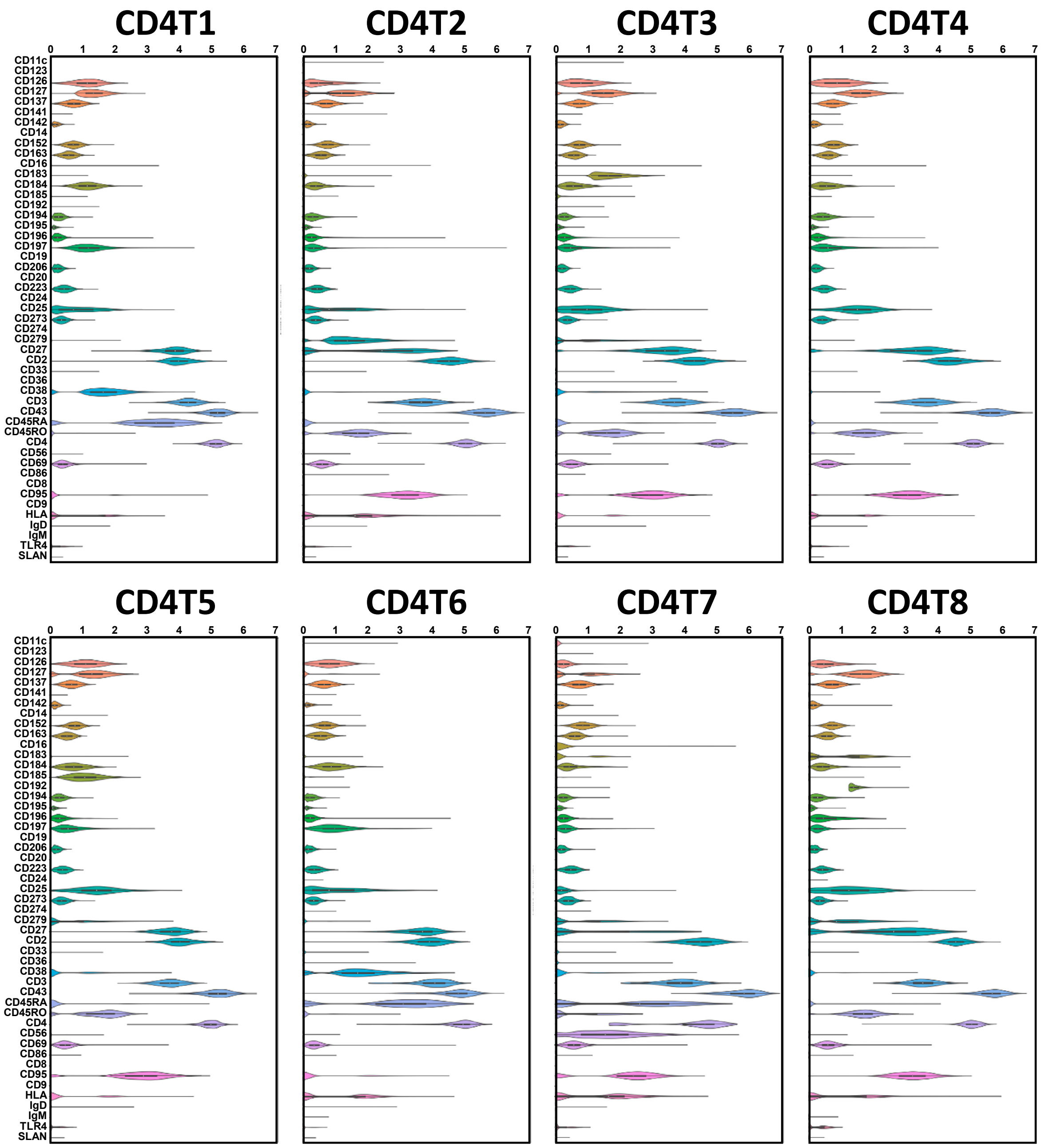
CD4T1
CD4T2
CD4T3
CD4T4
CD4T5
CD4T6
CD4T7
CD4T8
0 1 2 3 4 5 6 7
CD11c
CD123
CD126
CD127
CD137
CD141
CD142
CD14
CD152
CD163
CD16
CD183
CD184
CD185
CD192
CD194
CD195
CD196
CD197
CD19
CD206
CD20
CD223
CD24
CD25
CD273
CD274
CD279
CD27
CD2
CD33
CD36
CD38
CD3
CD43
CD45RA
CD45RO
CD4
CD56
CD69
CD86
CD8
CD95
CD9
HLA
IgD
IgM
TLR4
SLAN

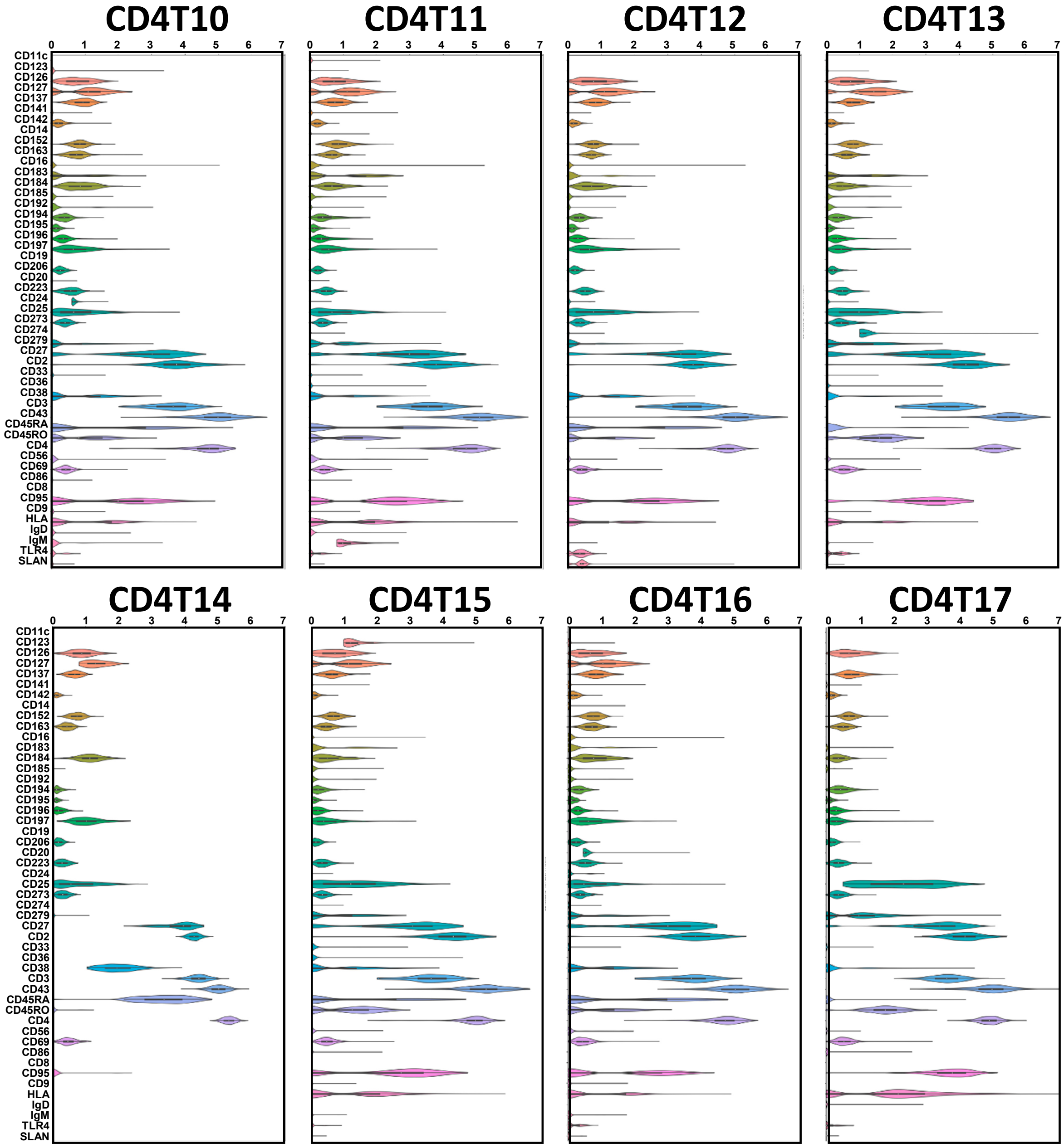

Supplemental Figure S2. Violin plots for surface marker expressions of each subcluster.

A. DEGs by CVD in CD4 subclusters

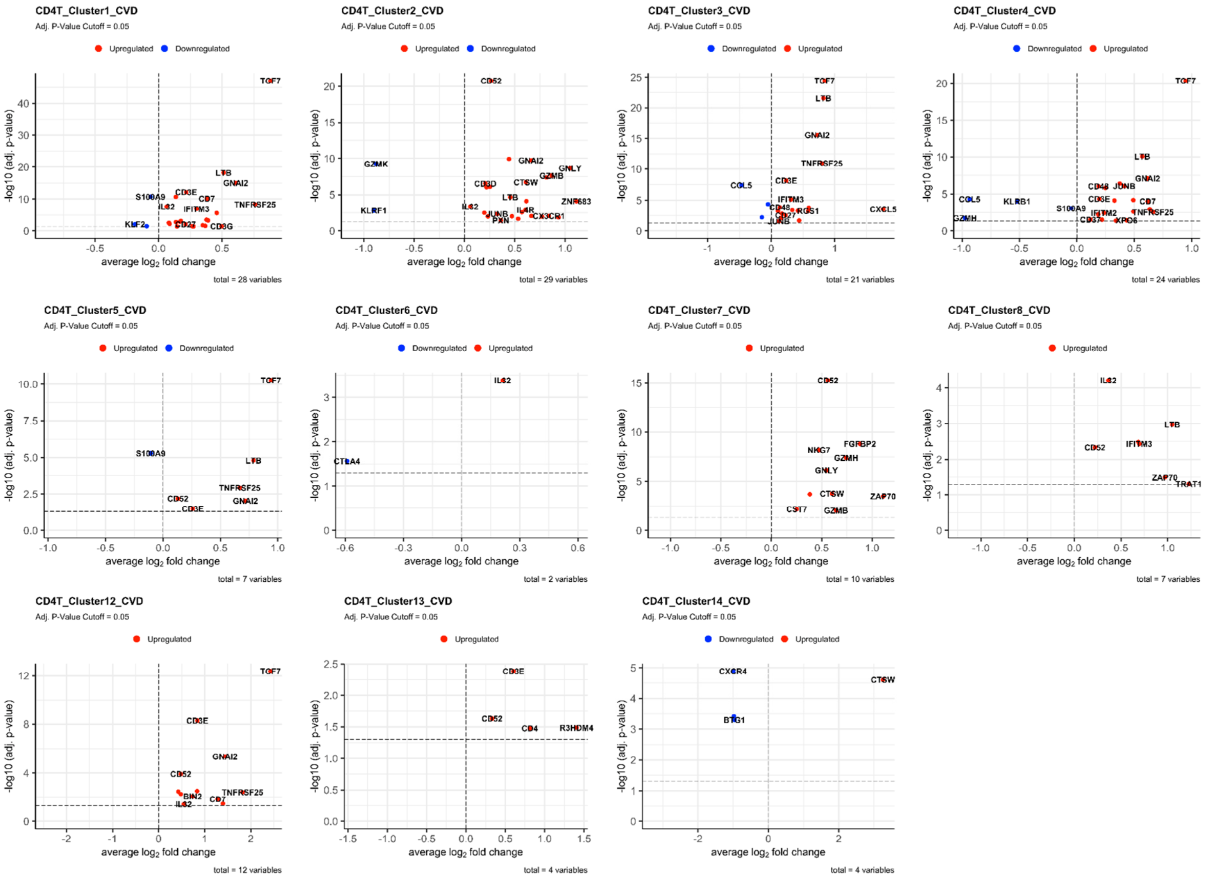

B. DEGs by Diabetes in CD4 subclusters

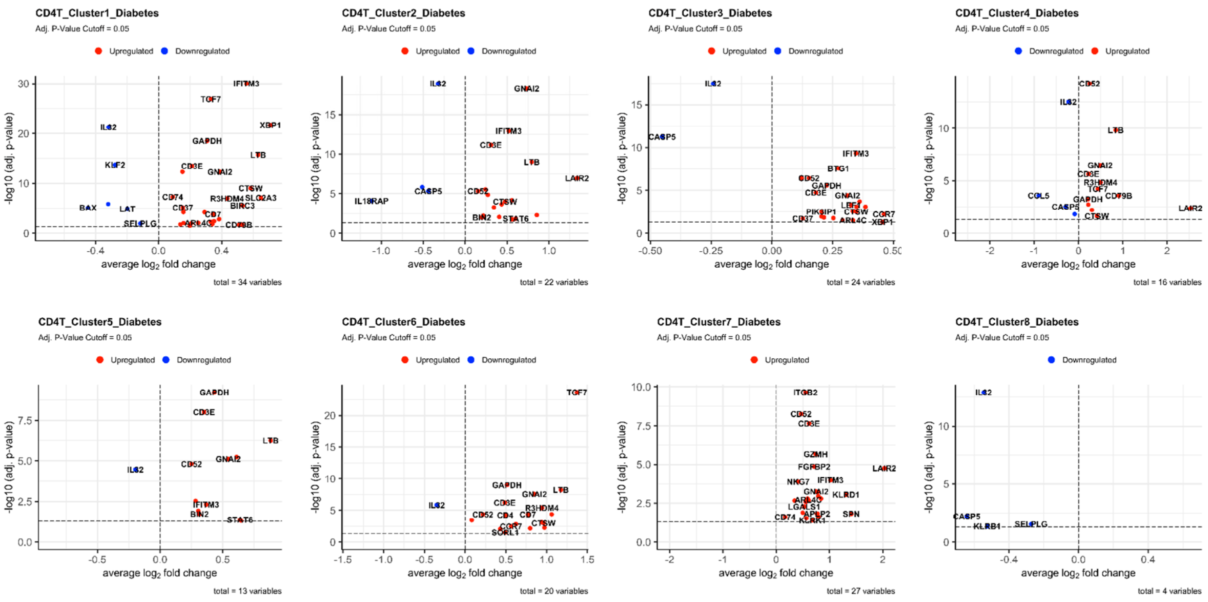

C. DEGs by Sex in CD4 subclusters

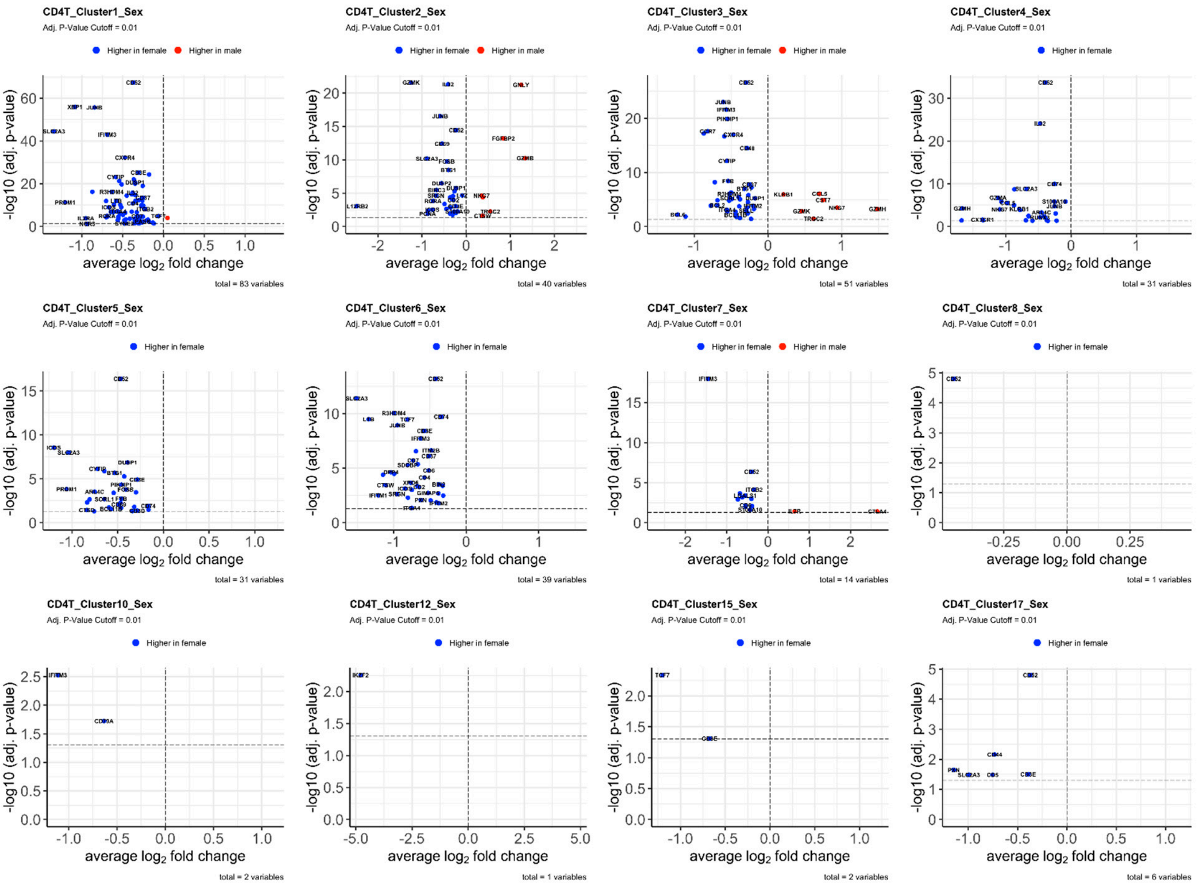

Supplemental Figure S3. Dotplots of differentially expressed genes between CAD+ vs CAD-, and between diabetes+ vs diabetes- in each CD4 T cell subcluster. The size of dots represents log(pct.1/pct.2). pct.1, the proportion of cells expressing each gene in Diabetes or CAD+; pct.2, the proportion of cells expressing each gene in Diabetes- or CAD-.

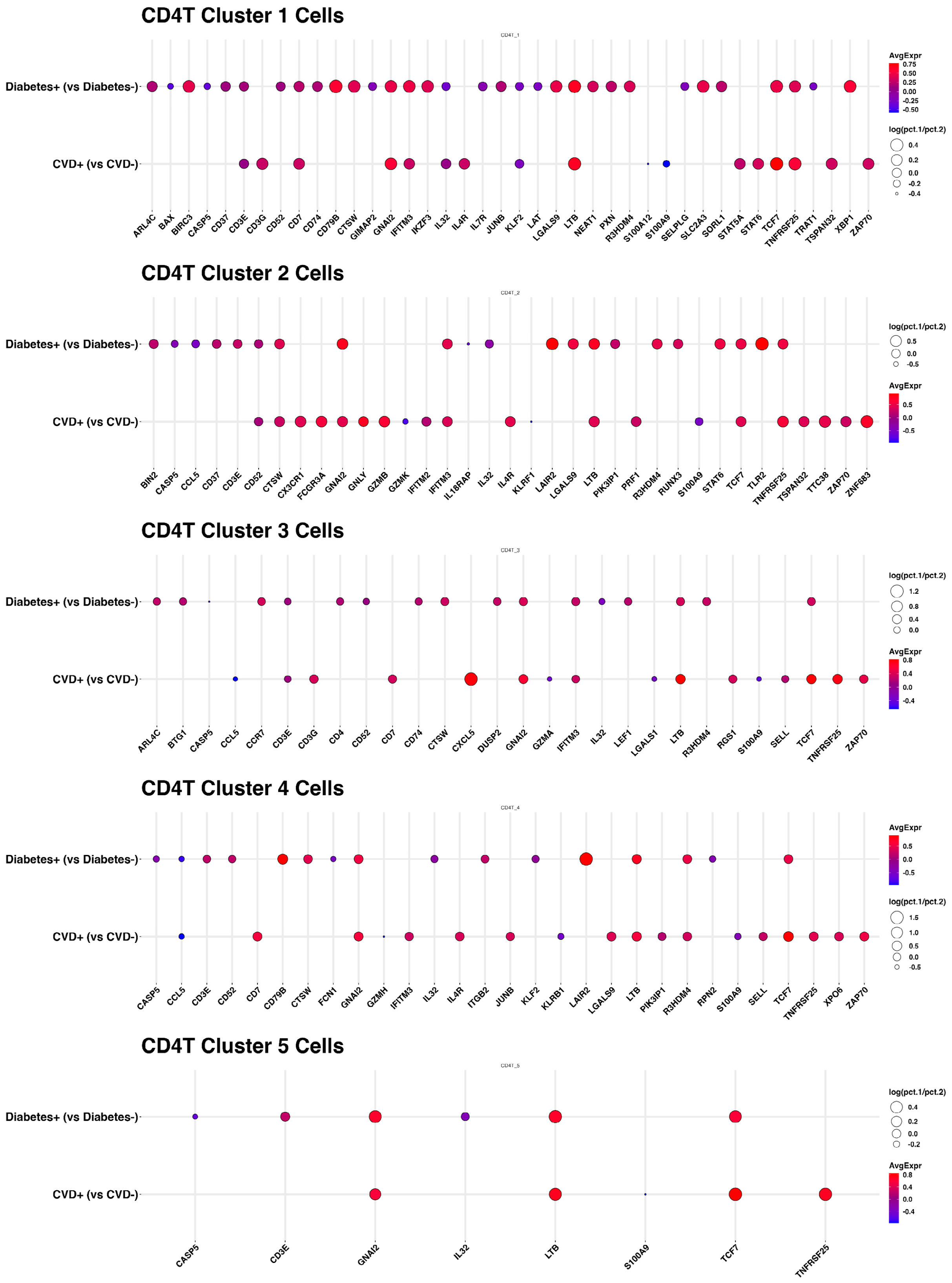
CD4T Cluster 1 Cells
CD4T_1
Diabetes+ (vs Diabetes-)
CVD+ (vs CVD-)
AvgExpr
0.75
0.50
0.25
0.00
-0.25
-0.50
log(pct.1/pct.2)
0.4
0.2
0.0
-0.2
-0.4
ARL4C BAX BIRC3 CASP5 CD37 CD3E CD3G CD52 CD7 CD74 CD79B CTSW GIMAP2 GNAI2 IFITM3 IKZF3 IL32 IL4R IL7R JUNB KLF2 LAT LGALS9 LTB NEAT1 PXN R3HDM4 S100A12 S100A9 SELPLG SLC2A3 SORL1 STAT5A STAT6 TCF7 TNFRSF25 TRAT1 TSPAN32 XBP1 ZAP70
CD4T Cluster 2 Cells
CD4T_2
Diabetes+ (vs Diabetes-)
CVD+ (vs CVD-)
log(pct.1/pct.2)
0.5
0.0
-0.5
AvgExpr
0.5
0.0
-0.5
BIN2 CASP5 CCL5 CD37 CD3E CD52 CTSW CX3CR1 FCGR3A GNAI2 GNLY GZMB GZMK IFITM2 IFITM3 IL18RAP IL32 IL4R KLRF1 LAIR2 LGALS9 LTB PIK3IP1 PRF1 R3HDM4 RUNX3 S100A9 STAT6 TCF7 TLR2 TNFRSF25 TSPAN32 TTC38 ZAP70 ZNF683
CD4T Cluster 3 Cells
CD4T_3
Diabetes+ (vs Diabetes-)
CVD+ (vs CVD-)
log(pct.1/pct.2)
1.2
0.8
0.4
0.0
AvgExpr
0.8
0.4
0.0
-0.4
ARL4C BTG1 CASP5 CCL5 CCR7 CD3E CD3G CD4 CD52 CD7 CD74 CTSW CXCL5 DUSP2 GNAI2 GZMA IFITM3 IL32 LEF1 LGALS1 LTB R3HDM4 RGS1 S100A9 SELL TCF7 TNFRSF25 ZAP70
CD4T Cluster 4 Cells
CD4T_4
Diabetes+ (vs Diabetes-)
CVD+ (vs CVD-)
AvgExpr
0.5
0.0
-0.5
log(pct.1/pct.2)
1.5
1.0
0.5
0.0
-0.5
CASP5 CCL5 CD3E CD52 CD7 CD79B CTSW FCN1 GNAI2 GZMH IFITM3 IL32 IL4R ITGB2 JUNB KLF2 KLRB1 LAIR2 LGALS9 LTB PIK3IP1 R3HDM4 RPN2 S100A9 SELL TCF7 TNFRSF25 XPO6 ZAP70
CD4T Cluster 5 Cells
CD4T_5
Diabetes+ (vs Diabetes-)
CVD+ (vs CVD-)
log(pct.1/pct.2)
0.4
0.2
0.0
-0.2
AvgExpr
0.8
0.4
0.0
-0.4
CASP5 CD3E GNAI2 IL32 LTB S100A9 TCF7 TNFRSF25

CD4T Cluster 6 Cells

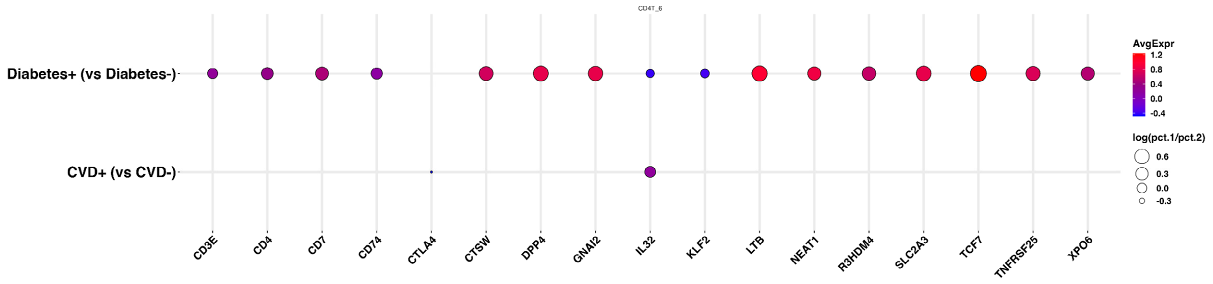

CD4T Cluster 7 Cells

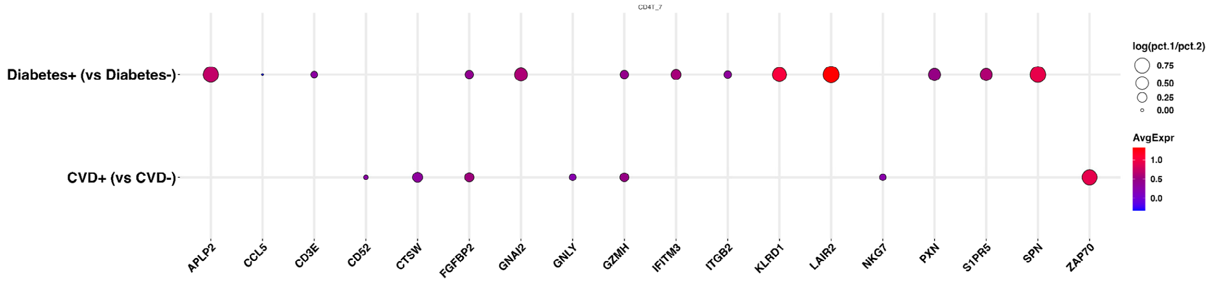

CD4T Cluster 8 Cells

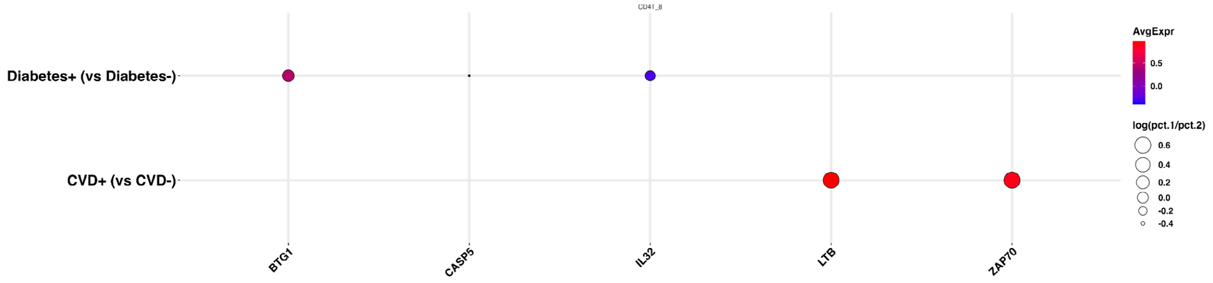

CD4T Cluster 11 Cells

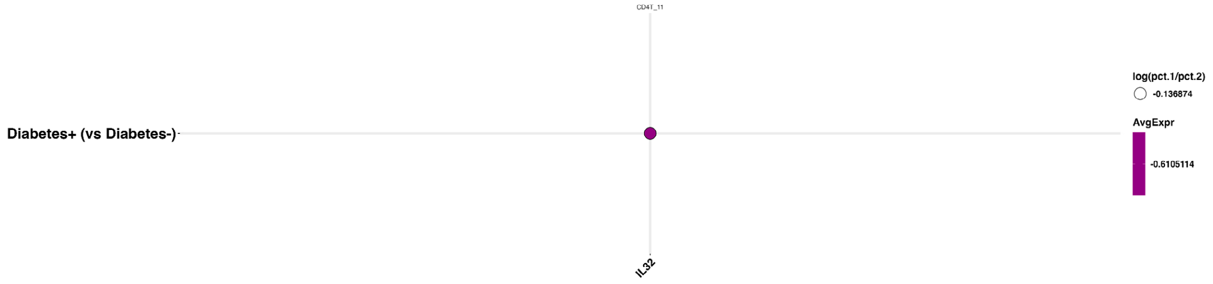

CD4T Cluster 12 Cells

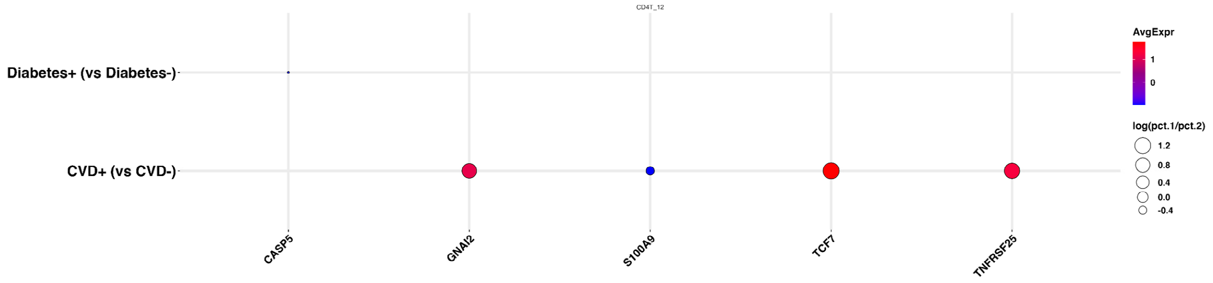

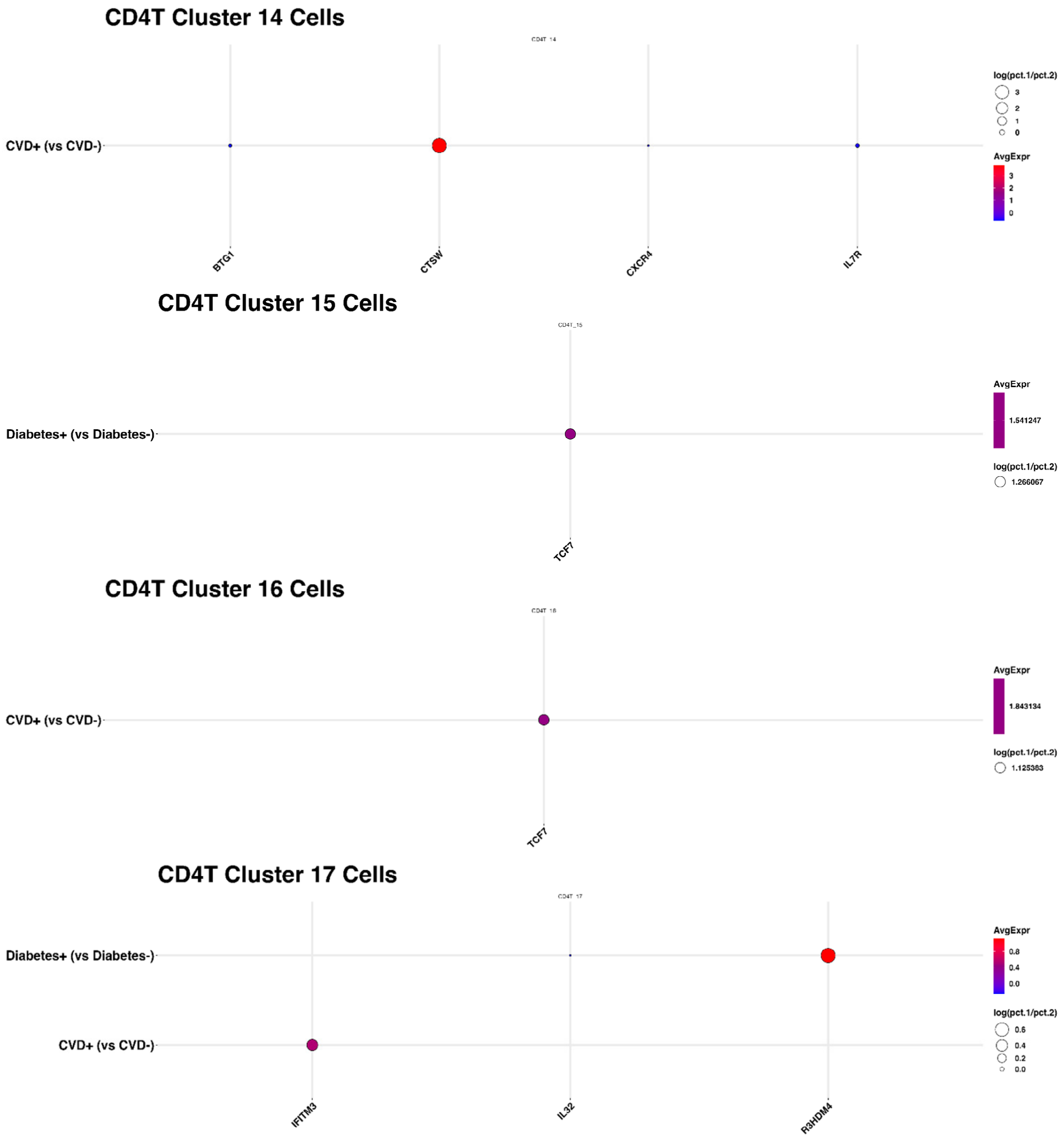

Supplemental Figure S4. A. Volcano plots for significantly upregulated genes by CAD in CD4 subclusters. B. Volcano plots for significantly upregulated genes by Diabetes in CD4 subclusters. Volcano plots for significantly upregulated genes by Sex in CD4 subclusters.

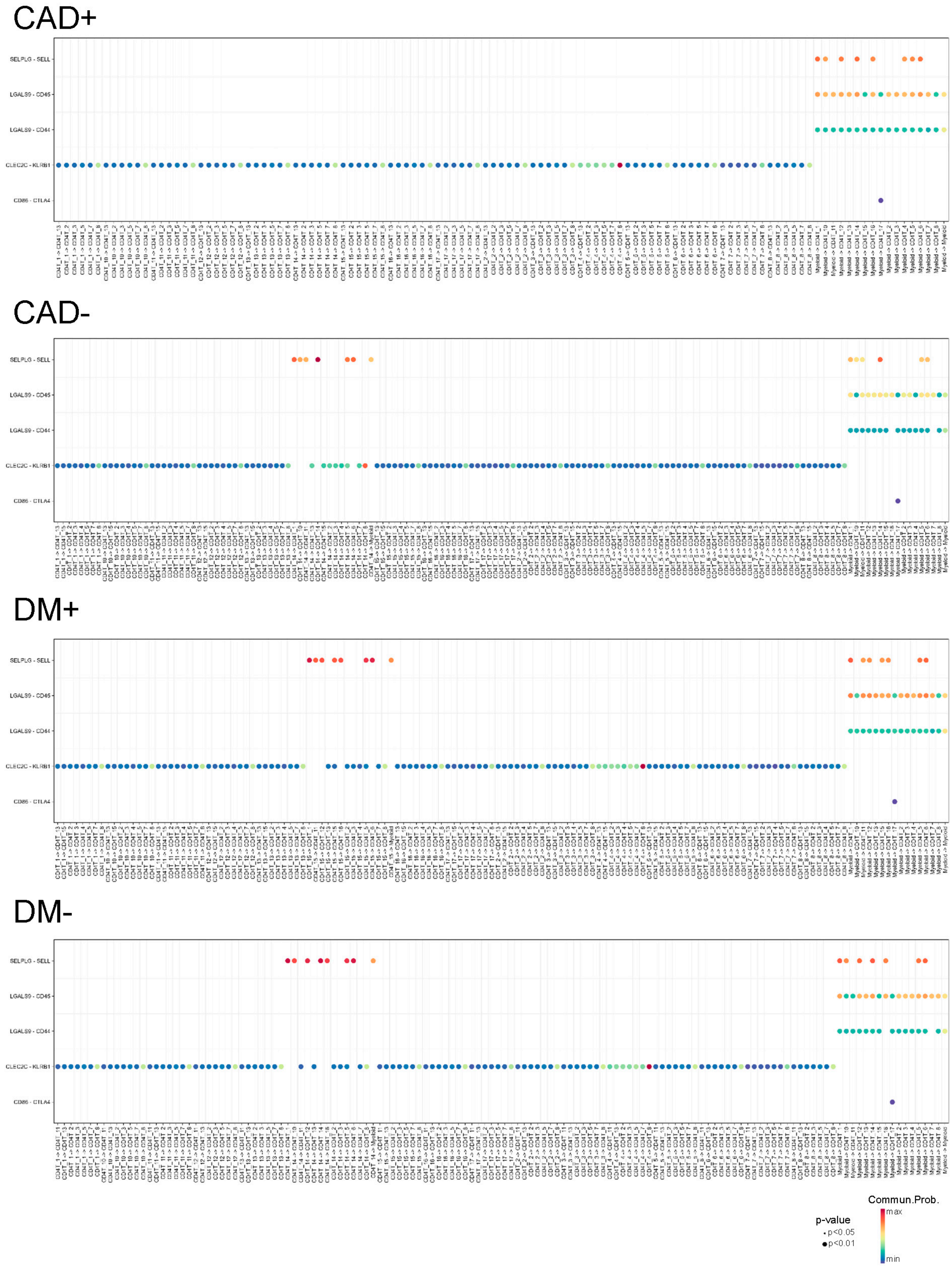
CAD+
CAD-
DM+
DM-
SELPLG - SELL
LGALS9 - CD45
LGALS9 - CD44
CLEC2C - KLRB1
CD86 - CTLA4
Commun.Prob.
max
min
p-value
p<0.05
p<0.01

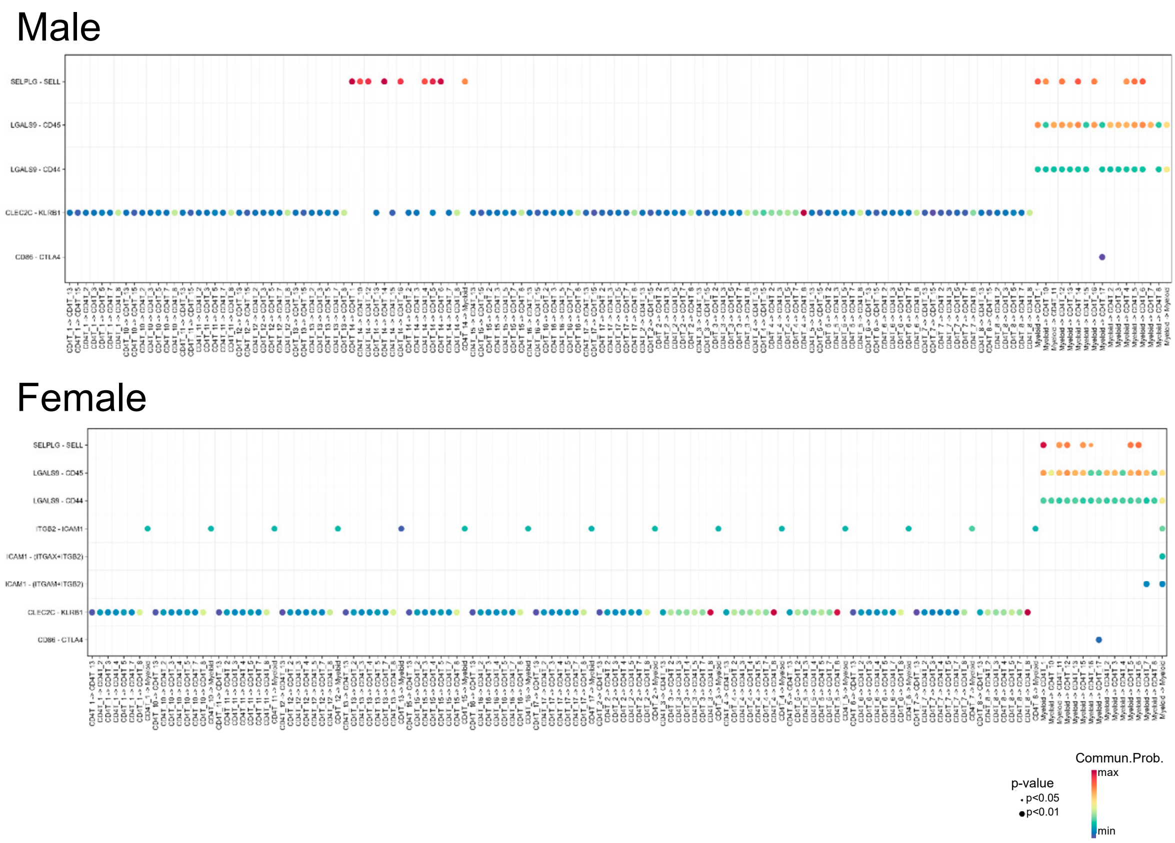

Supplemental Figure S5. Bubble plots of all interaction analysis between CD4T-CD4T cells and CD4T-myeloid cells with significant communication probabilities.

Supplemental Excel File S1. All the counts of gene expression in each single cell.

Supplemental Excel File S2. Differentially expressed genes in each cluster against all the others.

Supplemental Excel File S3. Differentially expressed genes by disease status in total CD4 T cells.

Supplemental Excel File S4. Differentially expressed genes by disease status in each CD4T subcluster.
